# Physiological properties of enkephalin-containing neurons in the spinal dorsal horn visualized by expression of green fluorescent protein in BAC transgenic mice

**DOI:** 10.1186/1471-2202-12-36

**Published:** 2011-05-07

**Authors:** Teruyuki Fukushima, Masayuki Tsuda, Takefumi Kofuji, Yuuichi Hori

**Affiliations:** 1 Department of Physiology and Biological Information, Dokkyo Medical University, School of Medicine, Kitakobayashi 880, Mibu, Tochigi 321-0293, Japan; 2 Radioisotope Laboratory, Kyorin University, School of Medicine, 6-20-2, Shinkawa, Mitaka, Tokyo 181-8611, Japan

## Abstract

**Background:**

Enkephalins are endogenous opiates that are assumed to modulate nociceptive information by mediating synaptic transmission in the central nervous system, including the spinal dorsal horn.

**Results:**

To develop a new tool for the identification of *in vitro *enkephalinergic neurons and to analyze enkephalin promoter activity, we generated transgenic mice for a bacterial artificial chromosome (BAC). Enkephalinergic neurons from these mice expressed enhanced green fluorescent protein (eGFP) under the control of the preproenkephalin (PPE) gene (*penk1*) promoter. eGFP-positive neurons were distributed throughout the gray matter of the spinal cord, and were primarily observed in laminae I-II and V-VII, in a pattern similar to the distribution pattern of enkephalin-containing neurons. Double immunostaining analysis using anti-enkephalin and anti-eGFP antibodies showed that all eGFP-expressing neurons contained enkephalin. Incubation in the presence of forskolin, an activator of adenylate cyclase, increased the number of eGFP-positive neurons. These results indicate that eGFP expression is controlled by the *penk1 *promoter, which contains cyclic AMP-responsive elements. Sections obtained from sciatic nerve-ligated mice exhibited increased eGFP-positive neurons on the ipsilateral (nerve-ligated side) compared with the contralateral (non-ligated side). These data indicate that PPE expression is affected by peripheral nerve injury. Additionally, single-neuron RT-PCR analysis showed that several eGFP positive-neurons in laminae I-II expressed glutamate decarboxylase 67 mRNA and that some expressed serotonin type 3 receptors.

**Conclusions:**

These results suggest that eGFP-positive neurons in laminae I-II coexpress enkephalin and γ-aminobutyric acid (GABA), and are activated by forskolin and in conditions of nerve injury. The *penk1*-eGFP BAC transgenic mouse contributes to the further characterization of enkephalinergic neurons in the transmission and modulation of nociceptive information.

## Background

Enkephalin-containing neurons are found in several areas of the central nervous system, including the dorsal horn of the spinal cord [1-3], where they play an important role in the transmission and modulation of nociceptive information [4].

The analgesic effect of intrathecally administered 5-hydroxytryptamine (5-HT) type 3 (5-HT_3_) receptor agonist 2-methyl-serotonin is attenuated by the opioid antagonist naloxone [5,6], suggesting that endogenous opiate-like substances may be involved in 5-HT-induced antinociception. Enkephalin-containing neurons in the superficial dorsal horn (SDH) of the spinal trigeminal nucleus in rats are innervated by 5-HT-containing fibers [7-9]. Furthermore, we have reported that enkephalinergic neurons express 5-HT_3 _receptors in the dorsal horn of the spinal cord [10]. These findings suggest that some antinociception is elicited by activation of 5-HT_3 _receptors in spinal dorsal horn neurons containing enkephalin.

Immunohistochemical investigations showed that several SDH neurons contain both enkephalin and γ-aminobutyric acid (GABA) [11-13]. We showed that activation of 5-HT_3 _receptors facilitates the release of GABA from some interneurons in SDH [14]. Thus, the antinociceptive action of 5-HT may depend on the release of both enkephalin and GABA by SDH interneurons that express 5-HT_3 _receptors.

The preproenkephalin (PPE) promoter contains cyclic AMP (cAMP)-responsive elements, and the expression of enkephalin is controlled by cAMP [15]. We observed that forskolin, an activator of adenylate cyclase (AC), affects the expression of enkephalin-green fluorescent protein (GFP) fluorescence in neurons in mouse spinal cord sections transfected with the GFP gene driven by the PPE promoter [16].

Peripheral nerve injury and tissue inflammation often induce a state of abnormal pain known as neuropathic pain, which includes hyperalgesia and allodynia [17]. Chronic constriction injury (CCI) of the sciatic nerve increases Met-enkephalin immunoreactivity in the spinal cord [18]. Increases in enkephalin have also been described in spinal cord injury [19], polyarthritis [20], electrical stimulation [21], and various other preparations, using immunocytochemistry and in situ hybridization histochemistry to detect changes in enkephalin. To develop a new, more efficient, tool for the analysis of enkephalin promoter activity and characterization of enkephalinergic neurons, we generated a bacterial artificial chromosome (BAC) transgenic mouse in which enhanced green fluorescent protein (eGFP) is expressed in enkephalinergic neurons under the control of the PPE gene (*penk1*) promoter.

## Results

### eGFP expression and endogenous enkephalin expression

**Figure 1:**
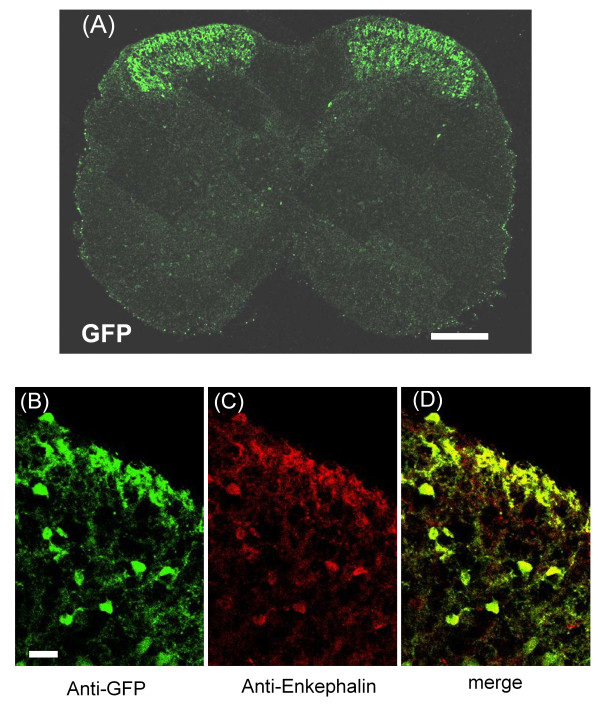
**Distribution of eGFP-positive neurons and double-staining IHC colocalization of eGFP expression with endogenous enkephalin**. (A) Low magnification of the L4 segment of the spinal cord shows the highest density of eGFP-positive somata and fibers in SDH. (B)-(D) Identification of eGFP-expressing neurons as enkephalinergic by double-staining IHC analysis with anti-eGFP (B, green) and anti-enkephalin (C, red) in SDH of *penk1*-eGFP BAC transgenic mouse. Scale bar = 200 μm in (A), 20 μm in (B)-(D).

Figure 1A shows an overview of the distribution of eGFP-positive neurons in the lumbar spinal cord, showing a dense distribution of eGFP-positive neurons in SDH and infrequent eGFP-positive neurons sparsely distributed throughout the deep dorsal horn and central canal. The observed distribution of eGFP-positive neurons is similar to that of a previous report demonstrating that many interneuronal somata in the substantia gelatinosa show enkephalin immunoreactivity [3,8,22].

To assess the correlation of eGFP fluorescence with endogenous enkephalin expression in SDH, we performed double immunostaining with anti-eGFP and anti-enkephalin antibodies. Figure 1(B-D) shows representative double-staining immunohistochemical images for eGFP and enkephalin. In 26 sections randomly selected from three mice, we determined that, of the 1550 lamina II neurons immunostained for eGFP, 1544 (99.6%) were also immunolabeled for enkephalin. In the same 26 sections, 1552 lamina II neurons were immunostained for enkephalin, and 1544 (99.5%) were immunolabeled for eGFP.

### Increased number of eGFP-positive neurons in the presence of forskolin

**Figure 2:**
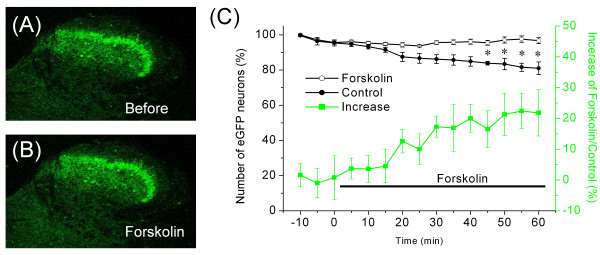
**Effect of forskolin on the number of eGFP-positive neurons**. (A, B) Representative images of eGFP fluorescence in the dorsal horn before (A) and after (B) treatment with forskolin. (C) Forskolin (5 μM) was applied as indicated by the horizontal bar. Data points on the graph (open circles and filled circles) indicate percentage of the number of eGFP-positive neurons normalized to the first ones at 10 minutes before forskolin treatment. Open circles and filled circles indicate the mean percentage of the number of eGFP-positive neurons with forskolin treatment (n = 7) and control neurons (n = 7), respectively. Filled squares indicate percent increases in the ratio of the number of eGFP-positive neurons with forskolin treatment to the number of control neurons. *P < 0.05 indicates a significant difference between the forskolin treatment and the control neurons.

The expression of enkephalin is controlled by cAMP, owing to the cAMP-responsive elements contained within the PPE promoter [23]. We examined whether our *penk1*-eGFP BAC transgenic mouse was useful for analyzing the effect of forskolin, an activator of AC, on the PPE promoter. To evaluate the effect of forskolin treatment, transverse spinal sections were exposed to a 60 minutes bath application of forskolin, and the number of eGFP-positive neurons was counted at 5-minute intervals. The number of eGFP-positive neurons in sections significantly increased compared to the control when forskolin was added to the Krebs solution, while the number of control (without forskolin treatment) decreased gradually with fading of eGFP fluorescence upon exposure to ultraviolet (UV) light, as illustrated in Figure 2C. The effect of forskolin was statistically significant (ANOVA, *P *< 0.05; Figure 2C) in a comparison between forskolin treatment (n = 7) and the control (n = 7). Percentage increase in the ratio of the number of eGFP-positive neurons with forskolin treatment to the number of control neurons was 21.8% ± 7.5% after 1 h of forskolin treatment (filled square in Figure 2C). This increase in eGFP-positive neurons may reflect an increase in the expression level of enkephalin above the detection threshold in neurons with relatively low basal enkephalin levels or alternatively, *de novo *enkephalin expression in neuron populations.

### Increased number of eGFP-positive neurons in partial sciatic nerve ligations

**Figure 3:**
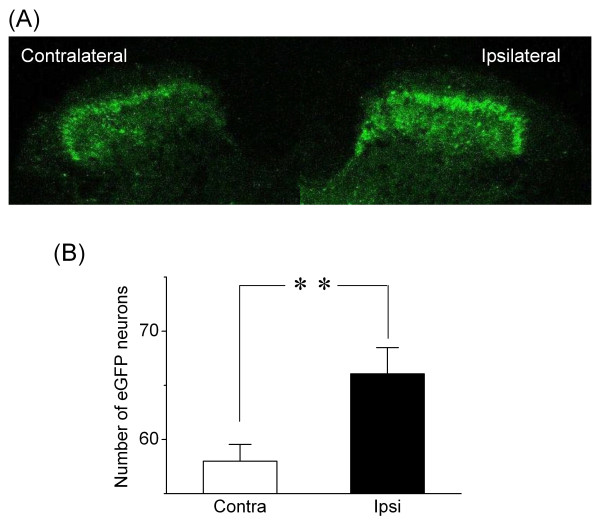
**Sciatic nerve ligation increased the number of eGFP-positive neurons**. (A) Representative image of eGFP fluorescence in SDH. eGFP fluorescence intensity was higher on the side ipsilateral (the right side of the figure) to sciatic nerve ligation 2 weeks after surgery compared with the contralateral (the left side). (B) Summary of the effect of nerve ligation on the number of eGFP-positive neurons. The number was the total of eGFP-positive neurons in an area of the SDH in an image scanned from a randomly selected section. Open bar indicates the side contralateral to ligation (n = 18); filled bar indicates the ipsilateral side (n = 18). **P < 0.01, significant.

Met-enkephalin immunoreactivity in the spinal cord dorsal horn increases after sciatic nerve ligation [18]. To confirm whether our BAC transgenic mouse is useful for analyzing the effect of sciatic nerve ligation on enkephalin expression, the number of eGFP-positive neurons on the sciatic nerve-ligated (ipsilateral) side was compared with the number on the nonligated (contralateral) side in spinal sections. The number of eGFP-positive neurons significantly increased in ipsilateral compared with contralateral [ipsilateral, 66.06 ± 2.42 (n = 18); contralateral, 58.00 ± 1.55 (n = 18); *P *< 0.01; Figure 3]. This result is consistent with a previous report [18].

### Reverse-transcription polymerase chain reaction (RT-PCR) analysis of the expression of glutamate decarboxylase 67 and 5-HT_3 _receptor mRNAs in eGFP-positive neurons

**Figure 4:**
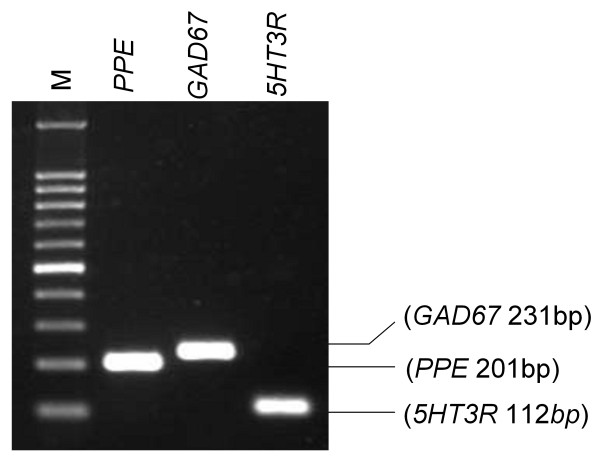
**Co-expression of PPE, GAD67 and 5-HT_3 _receptor mRNAs in an eGFP-positive neuron**. A neuron was subjected to RT-PCR detection of GAD67 and 5-HT_3 _receptor mRNAs. Lane M shows a 100-bp DNA ladder. Values in parentheses indicate the expected lengths of the PCR products for NSE and GAD67, 5HT3R, respectively.

PCR products of glutamate decarboxylase 67 (GAD67) and 5-HT_3 _receptor were detected in eGFP-positive neurons in SDH (Figure 4). The results of single-neuron RT-PCR indicated that GAD67 (detected in 15/15 eGFP-positive neurons, 100.0%) and 5-HT_3 _receptors (detected in 6/27 eGFP-positive neurons, 22.2%) were co-expressed in the somatodendritic regions of the enkephalinergic neurons in SDH.

## Discussion

### Distribution of enkephalinergic neurons in SDH

We visualized presumptive enkephalinergic neurons in the spinal cord sections by generating a BAC transgenic mouse in which eGFP is expressed in enkephalinergic neurons under the control of the *penk1 *promoter. Immunohistochemical investigations showed that enkephalinergic neurons were located in the dorsal horn of the spinal cord, with the highest density in the superficial laminae, thus suggesting eGFP-positive neurons are enkephalinergic. This distribution in the mouse model is similar to that described previously in rat and cat models [3,8].

### Co-expression of enkephalin, GAD67, and 5-HT_3 _receptor mRNAs in interneurons in SDH

Single-neuron RT-PCR analysis showed that most eGFP-positive neurons expressed GAD67 mRNAs and that some expressed 5-HT_3 _receptor mRNAs. We previously reported that enkephalinergic neurons express 5-HT_3 _receptors in SDH [10] and that activation of 5-HT_3 _receptors facilitates GABA release from some interneurons in SDH [14]. These results support the hypothesis that the release of enkephalin or the release of both enkephalin and GABA from some eGFP-positive neurons is facilitated by activation of 5-HT_3 _receptors.

### Changes in enkephalin expression induced by various treatments as models of chronic pain in the spinal cord

Analyses of the expression of enkephalin in the spinal cord during chronic inflammation have been previously reported in a number of models. Increases in PPE mRNA-labeled neurons in SDH have been reported in a number of injury model studies, including intraspinal microinjection of quisqualic acid to induce excitotoxic spinal cord injury [19], intraplantar injection of lambda carrageenan to induce hindpaw inflammation [24,25], and intradermal injection of heat-killed *Mycobacterium butyricum *to induce adjuvant arthritis [20]. In contrast to these inflammatory forms of chronic pain induced by various injections, there was no significant change in the spinal cord expression of enkephalin after sciatic nerve crush [18,26] or after complete transection of the sciatic nerve [25].

The impact of CCI, which produces a painful neuropathy of the sciatic nerve, is less clear. Quantification of immunohistochemical staining in the spinal cord dorsal horn showed an increase in Met-enkephalin [18,26]. On the other hand, no significant alteration in the level of PPE mRNA was detected by RNA blot analysis after CCI [25]. These reports suggest that even in the same chronic pain model, detection of enkephalin expression levels depend on the method of analysis used. Finally, in contrast to the results we report here, partial ligation of the sciatic nerve has been reported to produce a non-significant but moderate increase in enkephalin mRNA expression, as detected by *in situ *hybridization [27]. A possible explanation for these conflicting results is that the transgenic eGFP approach taken in our study is more sensitive than the *in situ *hybridization method.

### Possible causes for increased number of eGFP-positive neuron observed in sciatic nerve ligation or forskolin treatment

There are the two explanations for the increased number of eGFP-positive neurons: elevated expression of eGFP (and presumably enkephalin) above the detection threshold or new expression of eGFP (and presumably enkephalin) in populations that were previously eGFP-negative. The increased number of eGFP-positive neurons by sciatic nerve ligation or forskolin treatment may have resulted from both mechanisms, but in either case, it is impossible to determine which mechanism in this study. While our data does not provide direct evidence of a linear and homogeneous relationship between increased eGFP and enkephalin, this issue might be addressed in a comparison of the intensity of eGFP signal in neurons with low versus high enkephalin levels. If the comparison shows a precise relationship between increased eGFP and enkephalin, it might be possible to quantify how many treatments increase the number of enkephalinergic neurons and how much the expression level of enkephalin in each cell is increased.

### Other eGFP-transgenics used as tools in spinal dorsal cord studies

Figure 1 shows a dense distribution of eGFP-positive neurons in SDH. GABA-like immunoreactivity is present in 70% of met-enkephalin-positive neurons located in laminae II and III [13]. In transgenic mice expressing eGFP in GABAergic neurons under the control of the GAD67 gene (*GAD1*) promoter, almost every eGFP-labeled neuron in lamina II is GABAergic [28]. In addition, more than 80% of spinal neurons expressing GFP under the control of the prion promoter show GABA-like immunoreactivity [29]. Our RT-PCR analysis showed that almost all eGFP-positive neurons also express GAD67 (Figure 4). These data suggest that a subpopulation of eGFP-positive neurons in our study contains GABA in SDH.

Zeilhofer *et al*. generated BAC transgenic mice that express eGFP specifically in glycinergic neurons under the control of the glycine transporter GlyT2 promoter [30]. The BAC transgenic mice were used to measure the change in the number of spinal glycinergic neurons in CCI model of neuropathic pain [31]. The report suggests that loss of glycinergic neurons is not necessary for the development of pathological nociceptive behavior in the CCI model. GABAergic neurons containing enkephalin do not show glycine-like immunoreactivity [13]. The mechanism underlying the loss of spinal GABAergic neurons in neuropathic pain is still controversial, but this may be due to a combination of loss of neurons coexpressing GABA and glycine, and an increase in neurons coexpressing GABA and enkephalin.

## Conclusion

In conclusion, we generated BAC transgenic mice expressing eGFP in enkephalinergic neurons. These mice appear to be a feasible option for identifying enkephalinergic neurons under *in vitro *conditions in animal models of persistent pain (*i.e*., forskolin and sciatic nerve ligation). In addition, if eGFP is shown to have a linear relationship with enkephalin levels, this transgenic mouse may prove useful as an assay of enkephalin expression levels under various conditions both *in vitro *and *in vivo*. The physiological characterization of SDH neurons identified as enkephalinergic neurons would help elucidate the neuronal mechanisms underlying nociceptive modulation in the spinal dorsal horn.

## Methods

### Generation of penk1-eGFP BAC transgenic mice

To visualize enkephalin-containing neurons, we generated BAC transgenic mice expressing eGFP in enkephalinergic neurons under the control of the *penk1 *promoter. A BAC containing the *penk1 *gene was used to generate *penk1*-eGFP BAC transgenic mice. The eGFP gene was inserted after the ATG start codon of the *penk1 *gene. Transgenic mice expressing eGFP under the control of a BAC DNA containing the enkephalin gene were produced. BAC in *Escherichia coli *(*E. coli*) were modified based on a protocol developed by Yang *et al*. [32]. In the targeting construct, the eGFP cDNA was inserted in front of the stop codon of the *penk1 *gene, generating a C-terminal fusion protein. The recombinant cassette was introduced into pBluescript II KS (Stratagene), which contained the open reading frame of the eGFP gene. Fragment A (FA) consisted of 50 bp prior to the ATG site of the PPE gene, and fragment B (FB) consisted of 50 bp subsequent to exon 1 of the gene. pBluescript II KS-FA-eGFP-FB was transformed into DH10B *E. coli *containing BAC clone RP23-365K8 (Invitrogen, Carlsbad, CA, USA). Modified BAC clones were produced by two homologous recombinations in *E. coli *and confirmed by PCR analysis. The BAC DNA was prepared using a Nucleobond BAC 100 kit (Macherey-Nagel, Düren, Germany) and was linearized. Subsequently, the linearized DNA was run on a pulsed-field gel and purified from the gel with GenePure LE agarose (BM, Tokyo, Japan). The linearized BAC DNA was injected into pronuclei of C57BL/6 mouse zygotes. Genomic DNAs of founder mice were collected from their tail blood. The founder mice were screened by PCR for the presence of the eGFP gene and 5'- and 3'-ends of the linearized BAC DNA with primers 5'-GACACGCTGAACTTGTGG-3' and 5'-CTGGTCCTGATCCACGAC-3'. The founder mice were bred with wild-type C57BL/6 mice (Japan SLC, Shizuoka, Japan), F1 mice were subsequently crossed with each other, and then transgenic lines were maintained by sibling mating. In this study, these transgenic mice are referred to as *penk1*-eGFP BAC transgenic mice.

All animal experiments were approved by the institutional animal care and use committees at the Dokkyo Medical University. The care and use of the animals were in accordance with the National Institutes of Health guidelines on animal care and the guidelines of the International Association for the Study of Pain [33].

### Preparation of spinal cord sections

Animals aged 6-8 weeks were intraperitoneally anesthetized with pentobarbital (50 mg/kg) and segments of the spinal cord at the lumbosacral (L4-S1) level were removed. A microslicer (Dosaka EM, Osaka, Japan) was used to cut transverse sections in ice-cold modified Krebs solution [equilibrated with 95% O_2_-5% CO_2_, containing (in mM) 212 sucrose, 3 KCl, 25 NaHCO_3_, 1 NaH_2_PO_4_, 2 CaCl_2_, 1 MgSO_4_, and 11 D-glucose; pH 7.4]. The thickness of the sections was 350-450 μm for the measurement of fluorescence intensity of eGFP and 500 μm for the immunohistochemistry experiments.

### Immunohistochemical study

Transverse sections 500-μm-thick were stored in modified Krebs solution containing colchicine (10 μg/mL; Sigma, St. Louis, MO, USA) for 6 h at room temperature. Colchicine, an axonal transport blocking agent, was used to enhance the signal-to-background ratio and to clearly show the colocalization of endogenous PPE and eGFP in SDH neurons. The sections were cryoprotected in 30% sucrose/PBS overnight at 4°C. Cryosections of 20 μm thickness were prepared using a cryostat, and then blocked with 5% normal goat serum in PBS containing 0.1% Triton X-100 for 1 h at room temperature. After blocking, sections were immunostained with blocking solution containing mouse monoclonal anti-Met- and Leu-enkephalin antibody (1:200; Millipore, Billerica, MA, USA) and rabbit polyclonal anti-eGFP antibody (1:500; MBL, Woburn, MA, USA) overnight at 4°C. Sections were washed with PBS and then incubated with DyLight 488-labeled goat anti-rabbit IgG for anti-eGFP antibody and DyLight 549-labeled goat anti-mouse IgG (1:1000; KPL, Gaithersburg, MD, USA) for anti-enkephalin antibody for 1 h at room temperature. Sections were rinsed in PBS, mounted in Fluoromount (Diagnostic BioSystems, Pleasanton, CA, USA) and coverslipped. Sections were observed using a confocal laser scanning microscope (FluoView FV500; Olympus, Tokyo, Japan). The numbers of neurons immunolabeled with both anti-enkephalin and anti-eGFP as well as with anti-eGFP alone were counted.

### Measurement of eGFP-positive neurons in sections treated with forskolin

For these experiments and for single-neuron RT-PCR experiments, we used a fixed-stage upright microscope equipped with a confocal laser scanning system. Spinal sections were perfused with Krebs solution containing forskolin (an activator of AC; Sigma; 5 μM). To record the time course of eGFP fluorescence intensity, images were obtained every 5 min in the confocal laser scanning microscope. The percent increase was estimated by dividing the number of eGFP-positive neurons with forskolin treatment by the number of control neurons without forskolin treatment at the same time point.

### Partial ligation of the sciatic nerve

Partial ligation of the sciatic nerve was performed in *penk1*-eGFP BAC transgenic mice aged 7-9 weeks. The mice were kept at controlled room temperature under a 12 h light:dark cycle. They were anesthetized by intraperitoneal injection of sodium pentobarbital (50 mg/kg). The left sciatic nerve was partially ligated according to the protocol described by Seltzer *et al*. [34].

### Assessment of mechanical allodynia induced by partial ligation of the sciatic nerve

Each mouse was placed in a clear plastic box (height, 15 cm; diameter, 12 cm) on an elevated metal mesh floor. The withdrawal threshold to mechanical stimulation was determined. A mechanical stimulus was applied from underneath the mesh floor to the plantar aspect of the foot using an Electro von Frey (Model 1601; IITC, Woodland Hills, CA, USA). The lowest strength from five tests that induced a withdrawal response was considered the withdrawal threshold. Measurements were obtained on individual mice every day from 3 days prior to surgery to 21 days after surgery.

### Single-neuron RT-PCR

After incubation in modified Krebs solution for 1 h at 37°C, spinal sections were mounted in a recording chamber on the stage of a fixed-stage upright microscope (BX50WI; Olympus) and continuously perfused with Krebs solution [equilibrated with 95% O_2_-5% CO_2_, containing (in mM) 113 NaCl, 3 KCl, 25 NaHCO_3_, 1 NaH_2_PO_4_, 2 CaCl_2_, 1 MgCl_2_, and 11 D-glucose; pH 7.4].

After eGFP-positive neurons were identified in SDH using a confocal laser scanning system (FluoView 300; Olympus), as previously described [16], neurons were aspirated into a pipette under infrared differential interference contrast (IR-DIC) optics, and a CCD video camera (IR-CCD 2400; Hamamatsu Photonics, Hamamatsu, Japan) [10]. The collecting pipette had a tip diameter of approximately 3.5 μm and contained 2 μL of Ca^2+^-free and Mg^2+^-free PBS. The neurons were then injected into thin-walled autoclaved PCR tubes under gentle positive pressure and immediately frozen and stored at -80°C until use. The PCR tubes contained 2 μL MgCl_2 _(25 mM), 2 μL 10 × PCR buffer, 0.5 μL RNase inhibitor (40,000 units/mL), 2 μL nonionic detergent IGEPAL CA-630 (5%), and 5 μL diethylpyrocarbonate-treated water. On the following day, lysis was performed using IGEPAL CA-630 at room temperature for 5 min; the reverse transcription (RT) mixture, containing 1 μL oligo primers (0.5 μg/L), 2 μL mixed deoxynucleotide triphosphates (dNTPs, 10 mM), 2 μL dithiothreitol (0.1 M), 0.5 μL RNase inhibitor (40,000 units/mL), and 1 μL SuperScript II RT (200 units/μL), was then added. The reaction mixture was incubated at 42°C for 50 min and subsequently heat-inactivated at 70°C for 15 min. The total volume of 20 μL complementary DNA (cDNA) was stored at -20°C. PCRs were performed in 50 μL of solution containing 20 mM Tris-HCl, 50 mM KCl, 2.5 mM MgCl_2_, 0.2 mM dNTPs, and 2.5 units of Taq DNA polymerase. The concentration of primers was 20 nM in the first PCR and 200 nM in the second PCR. The primers targeted five genes: neuron-specific enolase (NSE), PPE, GAD67, 5-HT_3 _receptor, and eGFP. The primer sequences and product lengths are listed in Table 1.

**Table 1:** Primers used for single-neuron reverse transcription-polymerase chain reaction (RT-PCR)

Gene	Name	Primer Sequences	Sequence Start	Product Length, bp	GenBank Accession Number
*PPE*	PPE-F	5'-CTACAGGCGCGTTCTTCTCT-3'	273	201	NM_001002927
	PPE-R	5'-AGCAGCAAACAGGATGA-3'	561		
					
*GAD67*	GAD67-F	5'-CAAGTTCTGGCTGATGTGGA-3'	1616	231	NM_008077
	GAD67-R	5'-GCCACCCTGTGTAGCTTTTC-3'	1846		
					
*5HT3R*	5HT3-F	5'-CGTCAAGTCTGGTTTCCTTACC-3'	1794	112	NM_013561
	5HT3-R	5'-GCATCTATGCAAGATGATTTGC -3	1905		

*PPE*, preproenkephalin; *GAD67*, glutamate decarboxylase 67 gene; *5HT3R*, 5-hydroxytryptamine (5-HT, serotonin) type 3 receptor gene. Two 5-HT_3 _receptor subunits were cloned, namely subunit A (5-HT_3A_) and subunit B (5-HT_3B_). Although expression of 5-HT_3A _subunits alone yields functional 5-HT_3 _receptors, it has been suggested that 5-HT_3B _subunits modify the physiological and pharmacological properties of 5-HT_3 _receptors [35,36]. In this study we attempted to detect the 5-HT_3A _subunits. The primers were used for both the first and the second PCR amplification.

The amount of cDNA used for the first PCR varied from 3 to 7 μL and 1 μL of the first PCR product was used for the second PCR. A thermal cycler (GeneAmp 2400; Perkin Elmer, Waltham, MA, USA) was programmed for 35-40 cycles of 1 min of denaturation (94°C), 1 min of annealing (54-59°C), and 1 min of elongation (72°C). The second PCR products were visualized by electrophoresis on 2% agarose gel with ethidium bromide staining. All products were sequenced with dye terminator chemistry (Applied Biosystems, Foster City, CA, USA) and a DNA sequencer (Model 377; Applied Biosystems), and matched the published sequences. All reagents for the RT-PCR procedure, except the RNase inhibitor (Toyobo, Osaka, Japan) and IGEPAL CA-630 (Sigma), were obtained from Gibco/Invitrogen (Carlsbad, CA, USA).

### Statistical analysis

Data are presented as mean ± SE unless otherwise stated. Effects of sciatic nerve ligation on the number of eGFP-positive neurons were compared using a paired *t*-test. In addition, Student's *t*-test was used when appropriate. The data for the forskolin experiment were statistically analyzed by two-way analysis of variance (ANOVA), followed by *post hoc *multiple comparison (Tukey's test). *P *< 0.05 was considered significant.

## List of Abbreviations

**5-HT: **5-hydroxytryptamine; **5-HT_3_**: 5-HT type 3; **AC: **Adenylate Cyclase; **BAC: **Bacterial Artificial Chromosome; **CCI: **Chronic constriction injury; **cAMP: **cyclic-AMP; **eGFP: **Enhanced Green Fluorescent Protein; **GABA: **γ-aminobutyric acid; **GAD67: **Glutamate Decarboxylase 67; **GFP: **Green Fluorescence Protein; **NSE: **Neuron-Specific Enolase; **PPE: **Preproenkephalin; ***penk1: ***PPE gene; **RT-PCR: **Reverse-Transcription Polymerase Chain Reaction; **SDH: **Superficial Dorsal Horn;

## Authors' contributions

YH, TF and TK participated in the preparation of this manuscript and approved the final version of the manuscript. The individual contributions of the four authors to the manuscript are as given below. TF took the photographs and counted the number of eGFP-positive neurons on sections after the treatments. MT generated the BAC transgenic mice. TK carried out the immunohistochemical study. YH conceived the study and coordinated all experiments.
